# Exploring the Experiences of Children in Kinship Care in Scotland: A Sequence Analysis of Care Journeys, Characteristics and Outcomes

**DOI:** 10.23889/ijpds.v9i5.2601

**Published:** 2024-09-18

**Authors:** Joanna Soraghan, Robert Porter

**Affiliations:** 1 CELCIS, University of Strathclyde

## Objective and Approach

An increasing proportion of children in care in Scotland are formally living with extended family or friends – known as kinship care. To gain an enhanced understanding of experiences within kinship care, sequence analysis methods were applied to determine distinct patterns in care journeys throughout their first 5 years in care. Through linkage to education and health data, associations between these distinct types of care journey and children’s characteristics and outcomes were assessed.

## Results

Using a sample of 2987 children born between 2008 and 2014 who had experienced kinship care, we identified four ‘typical’ care journeys which best describe their experiences in terms of the type, number and duration of care placements. Our analysis found that children who entered care as infants were more likely to experience short-term kinship care, while those with a disability were more likely to spend extended periods in foster care than kinship care. Those living mainly in kinship care had poorer educational outcomes than those living mainly in foster care. 

## Conclusions

Sequence analysis can provide a more holistic view of children’s experiences over time than annual ‘snapshot’ data. The approach enabled us to identify different types of care journey, the characteristics of children who may be likely to have these experiences, and what their outcomes may be. 

## Implications

These findings have implications for the commissioning and design of support services for families who experience kinship care, and highlight the importance of consistent, long-term data collection to facilitate longitudinal analysis.

